# ADDRESSING THE EDUCATIONAL DISPARITIES IN COGNITIVE FUNCTION: THE COMPENSATION EFFECTS OF SOCIAL NETWORKS

**DOI:** 10.1093/geroni/igae098.0694

**Published:** 2024-12-31

**Authors:** Siyun Peng, Brea Perry

**Affiliations:** Indiana University Bloomington, Bloomington, Indiana, United States; Indiana University, Bloomington, Indiana, United States

## Abstract

Educational disparities have been widely considered a key driver of disparities in dementias and cognitive aging. In this study, we ask: Is it too late for less educated older adults to engage in cognitively stimulating environments (e.g., bridging social capital in networks) to shorten their cognitive gaps with more educated older adults? Bridging social capital is defined as access to novel information and is thought to be a source of cognitive stimulation that may confer resilience to cognitive decline. Using rich cognitive assessments and egocentric network data from the state representative Person to Person Health Interview Study (P2P; N=685), we find that the associations between bridging social capital and cognitive outcomes are strongest for people with less than a high school education. Importantly, the cognitive gaps among people with different educational levels disappear among those with higher bridging social capital. One important implication of this study is that stimulating environments in later life (e.g., bridging social capital) is most effective for the population with lower distal stimulating environments (e.g., people without a high school diploma). Finally, the relationship between bridging social capital and cognitive health is discussed in the context of cumulative stimulating environments across the life course.

